# Latino Veterans with PTSD: A Systematic Review

**DOI:** 10.3390/bs4030320

**Published:** 2014-09-03

**Authors:** James O. E. Pittman

**Affiliations:** 1 Department of Veterans Affairs Center of Excellence for Stress and Mental Health, 3550 La Jolla Village Drive, San Diego, CA 92161, USA; E-Mail: james.pittman@va.gov; Tel.: +1-858-552-8585 (ext. 7787); 2 Smith College School for Social Work, Lilly Hall, Northampton, MA 01063, USA

**Keywords:** Latino veterans, Hispanic veterans, minority veterans, posttraumatic stress disorder, Latino culture, Hispanic culture, PTSD and culture, review

## Abstract

Latinos have a long history of military service with recent service including combat conditions and multiple deployments, which are highly associated with posttraumatic stress disorder (PTSD). Clinical acumen underscores the importance of culture in assessment and treatment, but there has been little scientific literature that investigates the unique needs of veteran Latinos with PTSD. The primary goal of this systematic review was to analyze the existing literature on Latino veterans with PTSD and to critically evaluate attention to cultural issues. The Preferred Reporting Items for Systematic Reviews and Meta-Analyses were used to guide this review. Peer-reviewed, research reports written in English on Latino Veterans with PTSD since 1980 were included; 20 were assessment related, and nine were treatment related. All studies were quantitative. Only 13 studies mentioned culture as part of the context for Latino veterans, and only seven included cultural factors as part of the study design. Present findings highlight a lack of research focused on understanding cultural factors related to the assessment and treatment of Latino veterans with PTSD. Culturally-informed research on Latino veterans from current wars, Latina veterans and Latino veteran treatment outcomes are necessary to provide culturally-appropriate care to this growing veteran subgroup.

## 1. Introduction

Latinos are the fastest growing and the largest ethnic minority group in the United States, comprising 16.3 percent of the total population [1]. Latino, often used synonymously with the term Hispanic, broadly describes people with Latin American ancestral origin in the United States [2]. Latino, or Latina when referring specifically to women, are used herein for simplicity, respecting that individuals may prefer various other terms to describe their ethno-racial identity. Latinos are underrepresented in the U.S. Armed Forces, representing 12.3% of active duty service members, though accession and retention trends from 11.5% in 2003 to 16.9% in 2011 suggest the growth of this underrepresented group [3]. In 2010, there were 1.3 million Latino veterans in the United States, and a 23% increase is expected by 2030 [4]. Latinos represent 18% of the 2.1 million U.S. troops deployed to the Operation Enduring Freedom (OEF), Operation Iraqi Freedom (OIF) and Operation New Dawn (OND) wars [5] and, consequently, have been exposed to traumas that can result in conditions, like posttraumatic stress disorder (PTSD). In this context, the primary goal of this systematic review was to identify and analyze the existing literature on Latino veterans with PTSD focusing on the importance of culture.

### 1.1. PTSD in Veterans and Latinos

PTSD is a mental health condition that may occur after exposure to a traumatic event, such as combat [6], with lifetime prevalence rates reported up to 31% among Vietnam theater veterans and current prevalence up to 13% among OEF/OIF veterans [7]. The impact of PTSD on veteran functioning has been significant and wide-ranging, including decreased quality of life, increased risk of substance use disorders and suicide and increased physical health symptoms [8,9,10,11], but continued research to better understand this mental illness is warranted. Because PTSD has high rates and serious consequences among military combat veterans, significant efforts have been made to compile and organize the knowledge related to PTSD in veterans. For a comprehensively-elaborated compilation of the research on combat related PTSD, see the Institute of Medicine [5,12] or Tanielian and Jaycox [11]. The literature on PTSD and veterans has grown fast, but has rarely identified specific results related to Latino veterans.

Researchers and clinicians have asserted that cultural factors influence how people perceive and interpret their experience and can affect aspects related to understanding the development of PTSD [13,14,15,16] and that symptom expression is shaped by cultural influences [14,17]. A growing body of conceptual and research literature primarily focused on civilian Latinos with PTSD has demonstrated the importance of including cultural factors when considering assessment and treatment with this group [18,19,20,21,22,23,24,25,26]. For example, Latinos have been at greater risk for and more vulnerable to PTSD than non-Latino groups [20,27,28]. Further, cultural factors, such as wishful thinking and self-blame coping, low social support, perceived racism, expressive style and acculturation level, partially explained the higher prevalence of PTSD in Latinos [20,25,26].

#### 1.1.1. Latino Culture and PTSD Assessment

As with any broad cultural grouping, it is important to recognize that Latino culture is heterogeneous with disparities between and within various Latino groups. Not all cultural factors are shared by all Latinos or Latino subgroups. Still, cultural factors, such as acculturation, racism and beliefs, are paramount when assessing the risk of PTSD in Latino populations. Increased acculturation, the process of adapting to the language, knowledge and values of another cultural group, has been associated with greater risk for PTSD for Latinos [29]. Relatedly, experiences of racism and discrimination are important cultural factors for assessment [15,30,31], and they have been associated with PTSD in Latinos [26,32]. Fatalism, the belief that outcomes are predetermined, has been shown to be a risk factor for PTSD in Latinos [28,33,34].

Racial biases, including the language (Spanish *vs*. English) used by the clinician, can impact assessment [35,36], and assessment should include Latino-specific tools (e.g., The Short Acculturation Scale [37] or the Cultural Identity Scale [38]) for cultural aspects that are not often included in assessment batteries for PTSD [33,36]. Latinos experiencing PTSD may also report somatic complaints, such as pain or gastrointestinal distress [14,23,26,31,35,39], and they may describe feeling sad, nervous or angry [22]. Latinos may underreport stress and have higher levels of avoidance and numbing than non-Latino Whites [26,36]. These cultural factors are important to include in PTSD assessment with Latinos and may inform subsequent treatment.

#### 1.1.2. Latino Culture and PTSD Treatment

Cultural factors are also important in the treatment of Latinos with PTSD. Language can have a profound effect in treatment [23,33,35,40], including an impact on healthcare [21], treatment delivery [41], engagement [40] and adherence [33]. Recognition of the importance of family, *familismo*, and adequate family participation in treatment is well represented in the literature [23,33,35,40]. Spirituality is often a support for Latinos [23,35], but related beliefs can also act as a barrier to treatment [28]. As such, it is important to inquire into the health-related spiritual beliefs of Latinos for treatment consideration [23,33,35]. Latinos with PTSD are less likely to seek treatment than non-Latino Whites [42], and perceived lack of provider cultural competence is a barrier for some Latino veterans to seek psychological care [31].

Together, the assessment and treatment literature on Latinos with PTSD suggests the importance of including cultural factors in research and clinical work with this group. The extent to which culture is considered in PTSD research with Latino veterans is unclear.

### 1.2. Rationale and Objectives

Past research and literature has focused on either veterans with PTSD or the importance of culture in assessment and treatment of PTSD in Latinos. There is relatively little literature that specifically focuses on understanding the unique assessment and treatment needs of Latino veterans with PTSD. Prior review papers have focused on culture and general mental health or PTSD [15,16,19,43]; others have focused on specific aspects of civilian/veteran Latinos and PTSD [13,20,22]. One recent review specifically focused on the PTSD treatment seeking behavior of rural Latino veterans [31], yet none to date have systematically reviewed the scientific literature on Latino veterans with PTSD. To fill the gap, the existing literature on Latino veterans with PTSD was reviewed and analyzed with a critical focus on considerations associated with Latino culture. The objectives were to: (1) describe the characteristics of research articles on Latino veterans with PTSD; (2) identify the extent to which cultural factors were included; (3) synthesize the primary results of included articles; and (4) discuss gaps in the literature and needs for future research.

## 2. Method

This systematic review adhered primarily to the strategies outlined in the Preferred Reporting Items for Systematic Reviews and Meta-Analyses (PRISMA) [44], a 27-item checklist and four-phase flow diagram designed to improve the quality and transparency of systematic reviews. PRISMA is endorsed by a variety of organizations and journals and freely available online. Of the 27-item checklist, seven are not reported in this review, because they were directly related to meta-analyses (Items 13, 16 and 23) or primarily focused on outcome studies (Items 12, 15, 19 and 22) and not applicable to this review. The existing literature specifically focused on Latino veterans with PTSD is sparse; therefore, this review was intended to include all peer reviewed published quantitative and qualitative scientific results related to the topic.

### 2.1. Article Selection

An EBSCO Information Services database search engine, covering MEDLINE, PsycINFO, Social Science Abstracts, Social Work Abstracts and Academic Search Premier, was used to identify potential sources from 1980 to the present limited to peer-reviewed publications and the English language. Separate searches of the Published International Literature on Traumatic Stress (PILOTS), Scopus and Web of Science databases were also performed. Individual search terms, including general phrases, such as Hispanic, Latino, posttraumatic stress disorder, PTSD or veteran, were too broad, yielding 56,576, 32,089, 34,061, 32,017 and 103,472 potential sources, respectively; therefore, one combined Boolean/phrase search was used. Terms to capture Latinos (Latino, Hispanic, Chicano and minorities) were combined with terms to capture PTSD (PTSD, posttraumatic stress disorder, posttraumatic stress, post trauma stress) and the term veteran. Duplicates were automatically removed by the search engine, and the citations were exported to RefWorks citation manager. Since the PILOTS database is specific to traumatic stress, only the terms to capture Latino were combined with veteran. The date last searched was March 19, 2014. The manual search included a review of reference lists from other reviews related to PTSD and minorities, dissertations and conceptual articles related to Latinos and Latino veterans with PTSD. Unique references from the PILOTS search and the manual review were added to RefWorks list for screening.

Sources included in this review had to meet the following criteria: (1) quantitative or qualitative study reporting results related to Latino U.S. veterans with PTSD; (2) peer reviewed; (3) available in English; and (4) published since 1980. Dissertations were not considered to be peer-reviewed unless published in a peer-reviewed journal. The year 1980 was chosen as a cut-off because the Diagnostic and Statistical Manual for Mental Disorders (DSM) version three [45] was the first to include PTSD as a diagnosis.

### 2.2. Data Collection Process and Data Items

The author independently screened the titles and abstracts of all citations for applicability to the predefined inclusion criteria. Potential articles were removed from consideration when there was a clear indication that the article did not meet one or more of the inclusion criteria. Full text articles were screened for inclusion/exclusion criteria when sufficient information was not available in the title and abstract. The remaining eligible articles were sorted based on key findings into two primary categories: (1) assessment related; or (2) treatment related. The author independently reviewed the eligible articles, extracting data on study design, sample size, percent Latino (and specific Latino subgroups), percent women, data source/setting, veteran service era, PTSD measure, key findings and cultural considerations.

Articles categorized as assessment related included those that provided the rates and severity of PTSD in Latino veteran samples, as well as those with main findings on factors that relate to PTSD co-morbidity, symptom presentation or potential PTSD correlates. The treatment category included articles reporting treatment preferences, utilization of services, barriers or facilitators to treatment or treatment outcomes in Latino veteran samples. All articles were reviewed for Latino cultural considerations, such as attitudes or values, beliefs and behaviors or experiences. Examples of attitudes/values were *caballerismo* and *machismo*, wishful thinking, self-blame coping. Beliefs included a range of spirituality or health- and healing-related beliefs and preferences. Behaviors and experiences included expressive style, help-seeking, experiences of racial discrimination and acculturation.

The extracted characteristics from each of the included studies were organized and summarized in tabular form. Key results from included studies were synthesized based on their relation to assessment or treatment in order to provide a summary of extant research on Latino veterans with PTSD. The extent to which cultural factors were included or considered in the literature was assessed. Articles that included cultural factors in the study design as reflected in the aims or the design variables were categorized as having included cultural factors. Studies that did not include culture, but specifically mentioned culture as a factor in understanding Latino veterans with PTSD in the manuscript, were categorized as mentioned. Articles that did not include or mention culture at all were categorized as not mentioned. Review results were discussed in the context of literature on Latinos with PTSD, and knowledge gaps and areas for further research were identified.

## 3. Results

### 3.1. Article Selection

**Figure 1:**
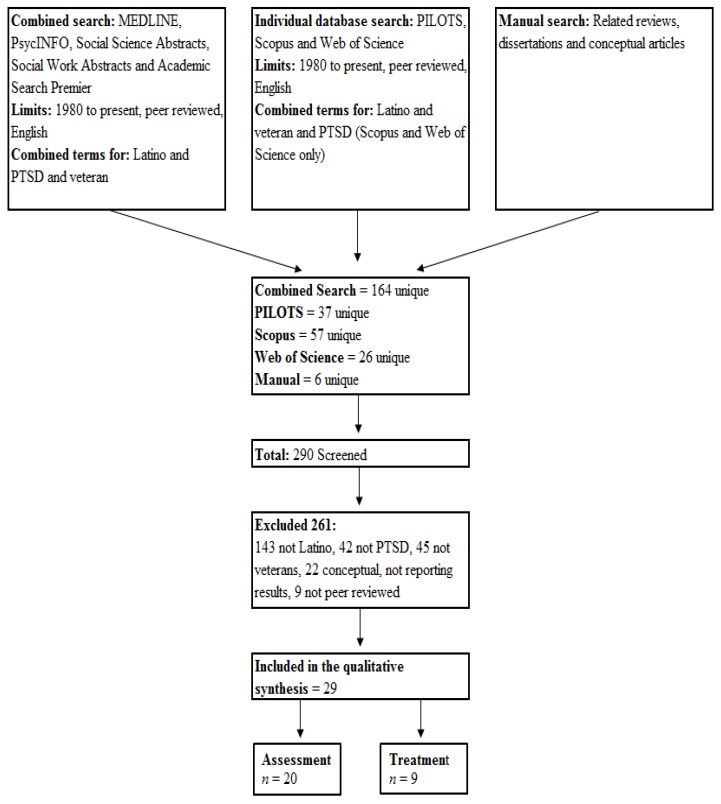
Flow diagram of article selection.

The combined EBSCO search covering MEDLINE, PsycINFO, Social Science Abstracts, Social Work Abstracts and Academic Search Premier using the combined phrases for PTSD, Latino and veteran yielded 164 unique potential articles. The PILOTS database search returned an additional 37 unique results. The Scopus and Web of Science databases respectively returned 57 and 26 unique potential articles, and the manual search yielded six. A total of 290 potential sources were screened for inclusion. Of those, 143 articles did not report results specific to Latinos, 45 did not report results related to veterans, 42 focused on mental health problems other than PTSD, 22 were conceptual articles not reporting research results and nine were not peer reviewed. The final sample included 29 articles (see Figure 1).

### 3.2. Article Characteristics

Table 1 summarizes the characteristics of the included articles. All eligible studies were quantitative and most were correlational (*n =* 24). Four studies had causal comparative designs, and one was a case study. Total sample sizes ranged from 18 to 732,085 veterans (4.2−100% Latino), with eight of the studies reporting a specific breakdown of Latino subgroups, including Cuban, Mexican American, Puerto Rican, South American or other South or Central American. Three studies included Latino women (Latinas); eight did not specify gender, but the majority of the studies (*n =* 18) did not include Latinas.

**Table 1:** Characteristics of the 29 articles on Latino veterans with PTSD.

Article by Category	Study Design/Type	Total Sample Size (% Latino)	% Latina	Latino Subgroup (% of Total Latino)	Veteran Era	Data Source (Setting)	PTSD Measure	Cultural Considerations
**Assessment Related**								
C'de Baca, Castillo and Qualls, 2012	Quan/Corr	398 (28)	28	Not reported	Unknown	VA New Mexico Women's Stress Disorder Clinic	CAPS	M
David *et al*. 1999	Quan/Corr	53 (17)	0	Not reported	Multiple	VA Miami Inpatient Unit	SCID III, MS	M
Dohrenwend *et al*. 2008	Quan/Corr	248 (33)	0	84 (MA (75), PR (18) O (7)	Vietnam	NVVRS	SCID	I
Escobar *et al*. 1983	Quan/Corr	41 (100)	0	Not reported	Vietnam	VA Los Angeles Clinic	DSM-III Symptom Checklist	I
Fontana and Rosenheck, 1994	Quan/Corr	1,198 (23)	0	Not reported	Vietnam	NVVRS	NVVRS Comp	NM
Ghafoori Heirholzer, 2010	Quan/Corr	96 (25)	0	Not reported	Unknown	Central California Vet Center	CAPS	M
Herrera, Owens, MallIkrodt, 2013	Quan/Corr	45 (100)	0	M (51), PR (27), CA (11), SA (7), C(4); O (13)	Unknown	Online Survey	PCL	I
Koopman *et al*. 2001	Quan/Corr	102 (11)	0	Not reported	Vietnam	VA Inpatient PTSD Program	CAPS	M
Kulka *et al*. 1990	Quan/Corr	1,198 (23)	Unknown	Not reported	Vietnam	NVVRS	NVVRS Comp	NM
Lewis-Fernández *et al*. 2008	Quan/Corr	255 (33.7)	0	MA (73); PR (20), O (7)	Vietnam	NVVRS	M-PTSD and SCID	I
Ortega and Rosenheck, 2000	Quan/Corr	1,195 (23)	0	MA (63), PR (23), C (2), CSA (3), O (9)	Vietnam	NVVRS	MS, NVVRS Comp	I
Penk *et al*. 1989	Quan/Corr	770 (12.8)	0	Not reported	Vietnam	VA Dallas Substance Program	MMPI with PTSD Finley’s PTSD Checklist	M
Ruef, Litz, and Schlenger, 2000	Quan/Corr	3,016 (48.9)	0	MA (63), PR (23), C (2), CSA (3), O (9)	Vietnam	NVVRS	MS	I
Schlenger *et al*. 1992	Quan/Corr	3,016 (48.9)	Unknown	MA (63), PR (23), C (2), CSA (3), O (9)	Vietnam	NVVRS	MS, MMPI, SCID	I
Schnurr *et al*. 2003	Quan/Corr	482 (9.5)	0	Not reported	Vietnam	NVVRS and HVVP	SCID	NM
Schnurr, Lunney, and Sengupta, 2004	Quan/Corr	482 (9.5)	0	Not reported	Vietnam	NVVRS and HVVP	SCID	NM
Tanielian *et al*. 2008	Quan/Corr	1,965 (8.3)	Unknown	Not reported	OEF/OIF	Random Sample of those Deployed to OEF/OIF	PCL	NM
Wilcox, Briones, and Suess, 1991a	Quan/Corr	59 (not reported)	0	Not reported	Multiple	VA El Paso PTSD Clinic	Dx	M
Wilcox, Briones, and Suess, 1991b	Quan/Corr	59 (61)	0	MA (77), PR (23)	Unknown	VA El Paso PTSD Clinic	Dx	M
Zatzick *et al*. 1994	Quan/Corr	225 (33)	0	Not reported	Vietnam	NVVRS	SCID	M
Bauer *et al*. 2013	Quan/Corr	732,085 (4.2)	Unknown	Not reported	Multiple	VA Austin Data Center	Dx	NM
Brinker *et al*. 2007	Quan/Corr	255 (46.3)	0	Not reported	Unknown	Southwest and Northeast U.S. Community	PCL	M
Greenawalt *et al*. 2011	Quan/Corr	148 (27)	Unknown	Not reported	OEF/OIF	VA Central Texas (3 Sites)	PCL	M
Jeffreys *et al*. 2013	Quan/Caus	263 (55.8)	Unknown	Not reported	Multiple	VA Texas Outpatient	CAPS, PCL, MINI PTSD	NM
Rosenheck and Fontana, 1996	Quan/Caus	5,475 (8.1)	Unknown	PR (56), MA (44)	Vietnam	VA PTSD Program (53 Sites)	SCID-III	M
Rosenheck and Fontana, 2002	Quan/Caus	12,447 (5.3)	0.06	Not reported	Vietnam	VA Inpatient PTSD Program (49 Sites)	MS, PC-PTSD	M
Spoont *et al*. 2009	Quan/Corr	20,284 (5)	Unknown	Not reported	Multiple	VA National Patient Care Database	Dx	M
Wanner, Long, and Tang, 2010	Quan/Case Study	1 (100)	0	Not reported	Vietnam	Large VA Medical Center	PCL	NM
Zappert and Westrup, 2008	Quan/Caus	18 (5.6)	5.6	Not reported	Unknown	Palo Alto VA Woman’s Trauma Recovery Program	PCL, Dx	NM

Note. Q = qualitative; Corr = correlational; Caus = causal comparative; MA = Mexican American; PR = Puerto Rican; SA = South American; C = Cuban; O = Other; SCA = South or Central American; NVVRS = National Vietnam Veterans Readjustment Study; HVVP = Hawaiian Vietnam Veterans Project; NVVRS Comp = diagnostic algorithm based on responses to Mississippi Combat-Related PTSD Scale, the SCID PTSD Module (Diagnostic and Statistical Manual for Mental Disorders (DSM) III-R) and the PTSD Scale of MMPI; MS = Mississippi Scale, CAPS = Clinician-Administered PTSD Scale; MMPI = Minnesota Multiphasic Personality Inventory PTSD Module PCL = PTSD Checklist, Dx = provider diagnosis; SCID = Structured Clinical Interview for DSM; PC-PTSD = VA Primary Care PTSD Screen; MINI PTSD = The Mini International Neuropsychiatric Interview; M = mentioned; I = included; NM = not mentioned; OEF = Operation Enduring Freedom; OIF = Operation Iraqi Freedom.

Over half of the 29 studies’ samples were drawn from the Department of Veterans Affairs (VA) inpatient or outpatient clinics or clinic data (*n =* 16). As ten of the remaining 13 studies analyzed data from the National Vietnam Veterans Readjustment Study (NVVRS), there were only four non-VA based samples represented. Relatedly, the combat era for the majority of the studies’ samples was Vietnam Era veterans (*n =* 16), with the rest being unknown (*n =* 6), multiple eras (*n =* 5) or Operation Enduring Freedom and Operation Iraqi Freedom (OEF/OIF) (*n =* 2).

The most commonly used tools to measure PTSD were The Structured Clinical Interview for DSM (SCID; *n =* 7) [46], the PTSD Checklist (PCL; *n =* 6) [47], diagnosis from clinician or chart (*n =* 5) or the Mississippi Scale for Combat Related PTSD (*n =* 5) [48]. Only four articles used the gold standard Clinician-Administered PTSD Scale (CAPS) [49,50,51,52,53].

### 3.3. Assessment Related (n = 20)

The majority of the articles related to assessment reported results specifically focused on PTSD prevalence and severity in Latino veterans (*n =* 12). Of these 12 studies, ten reported that Latino veterans have significantly higher levels than non-Latino White veterans [11,32,54,55,56,57,58,59,60,61]. Current PTSD prevalence estimates for Latinos in these studies ranged from 27%–33% for Latino veterans compared to 9%–15% for non-Latino White veterans. Two studies reported no significant difference between Latino and non-Latino White veterans [50,62]. Nine of the studies that reported significant differences used the same NVVRS data, either the full dataset or the diagnosed subset.

Several of the NVVRS studies accounted for risk factors, such as lifetime exposure to trauma and combat, and found that those factors did not account for the difference between Latinos and non-Latino Whites [32,55,56,57,58,59]; however, one NVVRS study found that controlling for pre-combat younger age, lower education, lower Armed Forces Qualification Test (AFQT) scores and higher exposure reduced current PTSD prevalence to non-significance between Latino and non-Latino White veterans [54].

Three studies found non-significant differences between Latinos and other groups when clinician-administered PTSD measures were utilized compared to checklists or other self-administered tools [50,54,57].

#### 3.3.1. Assessment Related Symptoms and Comorbidity

Six studies reported information on specific symptom differences for Latino veterans with PTSD. Latino veterans reported significantly higher intrusive symptoms [56,58], hyperarousal, guilt, avoidance [58] and psychotic symptoms associated with trauma [63,64] than non-Latino White veterans. Results related to dissociative symptoms were mixed, with one study finding greater symptoms in Latinos than non-Latino Whites [53] and another finding no differences between these two groups [65]. Finally, one study found that Mexican American Latinos had significantly lower numbing scores compared to non-Latino White veterans [58].

Only two studies reported results related to co-morbidities specific to Latino veterans with PTSD. One study focused on substance abuse and found that Latinos with PTSD were not more likely to have substance abuse than non-Latino White veterans with PTSD, but Latinos with high distress are more likely to have substance abuse than non-Latino White veterans with high distress [66]. The other study reported that PTSD and being Latino was associated with meeting criteria for one of the Cluster A personality disorders, paranoid, schizoid or schizotypal personality disorder [51].

#### 3.3.2. Cultural Factors

Cultural factors (e.g., acculturation level, bi-lingual interviewers, expressive style or Latino-specific gender role constructs) were included in seven, mentioned in eight and not mentioned at all in five of the assessment related studies. Five out of seven studies that included cultural factors did so to further examine PTSD disparities among Latino veterans in the NVVRS sample. Predisposing factors, such as family relationships and other psychosocial factors were not found to account for the elevated prevalence of PTSD in Latinos [59]. Differences in language fluency, feelings of being understood and respected, racism, readjustment or trauma exposure were also discounted as the explanation for higher prevalence in Latinos [32], as were acculturation level [58] and greater Latino expressiveness [57]. The differences in current PTSD prevalence between Latinos and non-Latino Whites in NVVRS were accounted for by younger age entering the military, lower education level and lower AFQT scores [54].

The other two studies that included cultural factors were the only two assessment-related studies that used samples composed entirely of Latino veterans to address cultural questions related to assessment in Latino veterans with PTSD [67,68]. The results of the first study indicated that Latino veterans with PTSD had more negative relationships with family than Latino veterans without PTSD [67]. Highly symptomatic Latino veterans, those who endorsed more than the group mean number of diagnostic symptoms, reported significantly smaller social networks and more negative emotion toward family than those with a low number of symptoms [67]. In the second study, traditional machismo views, defined as hyper-masculinity, restricted affect, chauvinism and aggression, correlated with higher PTSD symptoms [68]. Traditional machismo was compared with a separate, but related concept, *caballerismo*. *Caballerismo*, characterized by emotional connectedness, family nurturing, ethnic heritage pride and respectful conduct, was not associated with PTSD status or less psychological distress [68].

### 3.4. Treatment Related (n = 9)

The majority (*n =* 6) of the studies related to treatment of Latino veterans with PTSD were focused on access and utilization of services. Latino veterans with PTSD appear to access treatment at similar rates as non-Latino White veterans with PTSD [69,70]. Latino veterans with PTSD are more likely than non-Latino White veterans with PTSD to receive second-generation antipsychotics [71] and other psychotropic medication [72]. They were also more likely to have been treated in traditional long-term inpatient programs, be in treatment groups longer, spend more time in abreactive treatment modalities and be more satisfied with their treatment than non-Latino White veterans [72,73]. It is important to note that all of these studies drew samples from within the VA, not from the community.

The sixth study did not compare service use between Latinos and other groups and found that Latino veterans with combat-related PTSD may not seek treatment as often as those Latinos with non-combat-related PTSD [74]. This was the only study to use a community sample.

#### 3.4.1. Treatment Outcomes

Of the three remaining treatment studies focused on Latino veterans with PTSD, two reported treatment results for one Latino veteran each. One case study described using exposure, relaxation and rescripting therapy (ERRT) with a Latino man to reduce PTSD symptoms [75]. The second study reported individual results of one Latina, of 18 total participants, who received modified cognitive processing therapy (CPT) in a residential setting and had a clinically significant reduction in symptoms [76]. Finally, in a review of VA treatment data, prolonged exposure (PE) therapy or CPT both improved PTSD symptoms with large effect sizes, and Latino veterans did not differ significantly from non-Latino White veterans on response to the treatments, though specific outcomes for Latinos were not provided [52]. It is noteworthy that Latino-specific symptom improvement was not reported, but for all participants, PE and not CPT reduced average PTSD symptom levels to below the clinically significant cut-off of 50 for the PCL.

#### 3.4.2.Cultural Factors

None of the treatment-related studies included cultural factors in the design of the research. Five studies mentioned culture (e.g., culture can play an important role, not including cultural factors is a study limitation), and four studies did not mention culture at all. The three treatment outcome studies were among those that did not mention culture at all.

## 4. Discussion

The goal of this systematic review was to identify and critically analyze the existing literature on Latino veterans with PTSD with a specific focus on Latino cultural considerations. A total of 29 identified articles sparsely addressed assessment and treatment topics from prevalence to treatment outcomes, indicating a dearth of literature focused on this particular group. Only 13 studies mentioned culture as part of the context for Latino veterans, and only seven included cultural factors as part of the study design. This is particularly concerning given evidence that cultural factors are important to the assessment and treatment of Latinos with PTSD [18,19,20,21,22,23,24,25,26].

### 4.1. Overall Limitations and Gaps

There were several common limitations of the research on Latino veterans with PTSD. Latinas were not well represented among samples, studies did not utilize a longitudinal design or randomized control trials and primarily used treatment seeking samples. Another common limitation was that the studies mostly compared Latinos to non-Latinos rather than between Latino veteran subgroups or testing hypotheses in primarily Latino samples. The lack of evidence about Latinas is particularly concerning, because women are also a growing part of the veteran community [3]. Future research should consider the needs of Latinas with careful consideration to the intersection of gender, culture and PTSD [20]. The lack of rigorous qualitative studies related to Latino veterans with PTSD is surprising given that qualitative methodology is particularly well suited to obtain rich descriptions of phenomena in context [77], such as developing a model for race-related PTSD [78]. Future research should employ qualitative methods or mixed methods to develop a more nuanced understanding of Latino veterans’ experience in relation to trauma, PTSD, assessment and treatment.

### 4.2. Assessment Related

Research on the conditional risk of PTSD in Latinos suggest that Latinos, compared primarily to non-Latino Whites, are at greater risk for PTSD [20]. The studies in this review, specifically Latino veterans, are not as conclusive, yet do support the potential for greater risk. It is noteworthy that the two studies that did not find significant differences in PTSD between Latino and non-Latino White veterans used treatment-seeking samples for PTSD [50] or substance abuse [62]; whereas those that found differences used random community samples of veterans deployed to Vietnam (NVVRS) [56] and OEF/OIF [11]. Additional research comparing treatment-seeking *versus* non-treatment-seeking war veterans is needed to clarify differences between Latino and non-Latino White veterans.

Relatedly, results on PTSD-linked symptoms suggest that Latino veterans have higher intrusive, hyperarousal, guilt, avoidance and psychotic symptoms associated with trauma and lower numbing symptoms than non-Latino White veterans. Results regarding dissociative symptoms were inconclusive. The findings of higher intrusive and hyperarousal symptoms and lower numbing is discrepant to other Latino PTSD literature that suggest Latinos may underreport stress or have higher levels of avoidance and numbing than non-Latino Whites [26,36]. The heterogeneity of assessment tools used in the studies reviewed here may be a contributing factor to this inconsistency.

As pointed out previously [20], the interviewer-administered measures of PTSD may be less likely to support disparate PTSD rates between Latino and non-Latino White veterans [50,54,57], highlighting the role of the clinician in the assessment process. Cultural differences between clients and clinicians can impact assessment, because culture influences concepts of mental illness and symptom manifestation [14]. Since the studies herein utilized PTSD measures predominately based on the conceptual criteria outlined in the then current version of the DSM, it is possible that impairment or culturally-related symptom experience was not adequately captured. An alternate explanation, that clinician assessment adds greater objectivity, is also plausible. The standardized measures used in the studies also focus on PTSD as grounded in a fear-conditioning response as opposed to other views of PTSD that address relational aspects of trauma and moral injury, which may be more applicable to this group, given the Latino cultural focus on family and spirituality.

Future research should focus on examining differences between self-report and clinical interview assessments and to better understand how cultural differences between clinician and Latino veteran may influence PTSD assessment. More broadly, there is considerable support for the cross-cultural legitimacy of the DSM diagnosis of PTSD, but additional research and modifications to assessment criteria are also necessary [43]. For example, the current diagnostic definition of trauma, actual or threatened death, actual or threatened serious injury or actual or threatened sexual violence [6], does not necessarily include the ongoing experiences of racism and discrimination that can be a source of trauma for minorities [15,26,30,31]. Future research specifically examining the effects of racism and discrimination as related to PTSD symptoms and the intersections of culture, race and gender may help to inform changes to diagnostic criteria.

In the studies reviewed here, the NVVRS sample was used to explore cultural factors that may impact assessment and explain the higher rates of PTSD among Latino veterans. Factors such as family relationships, differences in language fluency, feelings of being understood and respected, racism, acculturation level and greater Latino expressiveness, which did not account for the difference, but a combination of greater trauma exposure, pre-combat lower age, lower education and lower test scores did. These findings appear contrary to the non-veteran Latino PTSD literature that suggests that higher acculturation [29], racism and discrimination [26] and language [35,36] increase the risk for PTSD in Latinos. One possible explanation for the discrepancies between the findings in this review and those of non-veteran Latinos is the potential impact of military service on Latino cultural identity. Some evidence suggests that military service itself is associated with greater acculturation [79], perhaps reducing the impact of Latino-specific cultural factors on PTSD. Another potential explanation is that cultural factors and experiences with racism were not adequately assessed.

Further research is necessary to assess differences between veteran and non-veteran Latinos with PTSD. Given the demographic shifts since the NVVRS data were collected, future research should also focus on identifying cultural factors related to PTSD in OEF/OIF Latino veterans. The majority of the research to date has focused primarily on identifying differences of Latinos compared to non-Latino White veterans and explaining those differences. Future research on Latino veterans with PTSD should focus on identifying the specific needs of Latino veterans as a separate group. Only two studies in this review focused on such factors [67,68]. Longitudinal research is particularly necessary to determine cultural factors that are important risk, as well as resilience factors for Latino veterans to develop PTSD.

### 4.3. Treatment Related

Findings on Latino veteran treatment utilization were mixed. Latino veterans with PTSD seek treatment at the VA at similar rates as non-Latino White veterans [69], but outside the VA, Latino veterans with combat-related PTSD seek treatment less often than Latino veterans with non-combat PTSD [74]. Non-veteran-specific research suggests that Latinos generally underutilize mental health services [40] and Latinos with PTSD are less likely to seek treatment than non-Latino Whites [42]. Perhaps some aspect of veteran-specific care that the VA offers, such as low cost, could explain this difference. Still, others have asserted that Latino veterans have barriers to seeking care, such as racism, lack of cultural competency at the VA and resistance to access help outside the family [31]. The articles in this review showed that Latino *versus* non-Latino White veterans were more likely to receive second-generation antipsychotics or other psychotropic medication, be treated in inpatient PTSD programs, be in treatment groups longer, spend more time in abreactive treatment modalities and be more satisfied with their treatment than non-Latino White veterans [72,73]. Some of these results seem consistent with other literature on Latinos and may have cultural implications. For example, higher rates of inpatient PTSD treatment could be related to cultural factors, such as machismo. Latinos may see mental illness as a weakness and, therefore, wait to seek help until symptoms are very severe [40]. Latino veterans may respond to directive treatments rather than inner exploration [35], which could explain the differences in medication use and why Latino veterans would stay in treatment longer.

Additional research on Latino veteran PTSD treatment utilization of VA and non-VA services is needed to understand potential parity issues. Future research on utilization should examine treatment preferences for Latino veterans, as well as cultural factors that may affect those preferences. Potential PTSD treatment facilitators and barriers, such as stigma, provider cultural competence, machismo, familismo and treatment beliefs, should also be further explored.

The three articles specifically on the outcomes of treatment for PTSD in Latino veterans provided some preliminary support for ERRT, CPT and PE in Latino veterans [52,75,76], consistent with the other results from one community-based randomized controlled trial using a culturally-adapted version of CPT for PTSD in Latinos [80]. These results were also consistent with the conceptual literature suggesting that Latinos may respond well from directive approaches to treatment [35], because both PE and CPT are directive in that they include psychoeducational components and require regular completion of practice assignments [81,82]. Still, additional support for the efficacy and effectiveness of these treatments with Latino veterans is needed. Since culturally-cognizant theorists consistently assert that treatment of Latinos with PTSD should incorporate cultural factors, such as language, familism, spirituality and machismo [14,17,23,35,41,42,83,84,85], it is important that further treatment research with Latino veterans use culturally-modified approaches, such as Hinton *et al*. [43] and Kichic *et al*. [41].

Additional research is also needed on the efficacy and effectiveness of other PTSD treatment modalities, such as eye movement desensitization reprocessing (EMDR) [86] or psychodynamic approaches [87,88]. Further, cultural factors among those discussed above should be systematically studied to determine their effect on these treatments. Finally, future treatment outcome research should be focused on the effects of cross-cultural treatment and on understanding how to navigate cross-cultural issues in treatment with Latinos [14,41] and how non-Latino clinicians can prepare themselves for work with Latino veterans with PTSD [83]. Research on the impact of cross-cultural clinician training and education and their subsequent impact on treatment outcomes is also needed.

### 4.4. Limitations

This systematic review has several limitations. As with any review, articles meeting inclusion criteria may have been missed despite using multiple databases and a manual search. The use of only one researcher in the article screening and selection process is also a limitation. The diversity of articles included in this review prevented including a meta-analysis. The review criteria did not include studies on active duty service members or reservists, which may have enriched the results on Latinos currently serving in the armed forces, though not technically veterans.

## 5. Conclusions

Conceptual articles asserting the importance of culture on PTSD assessment and treatment in Latino veterans are not new [35,36], yet 15 to 20 years later, research dedicated to understanding the assessment-related resilience and risk factors for developing PTSD and the related concomitants is limited. Culturally-appropriate treatments for PTSD are beginning to emerge, but considerably more research is necessary to understand the treatment needs of the growing subgroup of Latino veterans.
